# Continuous intraperitoneal insulin infusion as a valuable approach in patients with unstable type 1 diabetes: two case reports and a mini review of the literature

**DOI:** 10.3389/fmed.2025.1657069

**Published:** 2025-09-08

**Authors:** Erika Pedone, Amelia Caretto, Giulio Belfiori, Anna Zanardini, Sergio Di Molfetta, Luigi Laviola, Francesco Giorgino, Lorenzo Piemonti, Marina Scavini, Andrea Laurenzi

**Affiliations:** ^1^Diabetes Research Institute, IRCCS San Raffaele Scientific Institute, Milan, Italy; ^2^Division of Pancreatic Surgery, Pancreas Translational and Clinical Research Center, IRCCS San Raffaele Scientific Institute, Milan, Italy; ^3^Department of Precision and Regenerative Medicine and Ionian Area, Section of Internal Medicine, Endocrinology, Andrology and Metabolic Diseases, University of Bari Aldo Moro, Bari, Italy; ^4^Unit of Regenerative Medicine and Organ Transplants, IRCCS Ospedale San Raffaele, Milan, Italy; ^5^University Vita-Salute San Raffaele, Milan, Italy

**Keywords:** continuous intraperitoneal insulin infusion, hypoglycemia, subcutaneous insulin resistance, glycemic variability, type 1 diabetes

## Abstract

In recent years, continuous intraperitoneal insulin infusion (CIPII) has become a valid therapeutic option to achieve good glycemic control for patients with unstable type 1 diabetes and subcutaneous insulin resistance, mainly due to the absorption of insulin through the portal venous system. This route improves hepatic uptake and reduces peripheral plasma insulin levels, also optimizing glucagon secretion and hepatic glucose production. CIPII can lead to better blood glucose control and more predictable insulin profiles, especially after meals, compared to subcutaneous injections. Therefore, some studies suggest that CIPII may reduce the risk of hypoglycemia compared to subcutaneous insulin as well as improving patient satisfaction. Actually, among CIPII delivery systems, DiaPort particularly stands out for its low side effects, proven clinical efficacy, and potential for integration into closed-loop systems.

## 1 Introduction

Continuous intraperitoneal insulin infusion (CIPII) is an option for insulin-treated patients with impaired subcutaneous insulin absorption and/or extreme swings in blood glucose despite optimal diabetes management. In patients with type 1 diabetes (T1D), CIPII improves glucose control without increasing the risk of severe hypoglycemia, possibly through restoring the physiological porto-systemic insulin gradient (1).

The DiaPort system is a commercially available system for CIPII. It is surgically implanted in the abdomen and insulin infusion is maintained using an external insulin pump, similar to those for continuous subcutaneous insulin delivery (2). The only insulin approved for the DiaPort system is Insuman Infusat® (Sanofi), since other insulins may precipitate resulting in early cannula occlusion. CIPII mimics physiology more closely than standard therapies, as multiple daily insulin injections (MDI) and continuous subcutaneous insulin infusion (CSII), through the subcutaneous route because most of the insulin is absorbed through the portal venous system. CIPII may be a therapeutic option in patients with severe subcutaneous (SC) insulin resistance, poor glycemic control with high daily insulin requirements, severe hypoglycemia and hypoglycemia unawareness during SC insulin therapy, skin disorders, SC site issues, lipohypertrophy and lipoatrophy (3).

Several clinical trials and observational studies have shown a decrease in glycated hemoglobin (HbA1c) and a lower incidence of hypoglycemia using CIPII, also reducing fasting insulin levels, in comparison with subcutaneous insulin delivery through CSII and MDI (4, 5). Furthermore, CIPII has been shown to increase patient satisfaction and quality of life (QoL) (6).

Here we present a short review on CIPII and we report the experience of our Center on the use of CIPII in two patients with unstable diabetes, focusing on glycemic outcomes during the use of the CIPII and during the period of discontinuation of CIPII due to Insuman Infusat® unavailability.

## 2 Case presentations

Patient 1 is a 47-year-old Caucasian woman diagnosed with type 1 diabetes since the age of 9 years and with a very unstable glucose control. Her diabetes was complicated by proliferative retinopathy treated with laser-therapy, nephropathy with proteinuria, peripheral sensory neuropathy, and autonomic neuropathy leading to urinary retention requiring self-catheterization, as well as severe slowing of the intestinal transit-time, causing several hospitalizations for intestinal subocclusion.

She also had a history of congenital ventricular septal defect not surgically corrected, hypertension treated with angiotensin converting enzyme (ACE)-inhibitors, beta-blockers and diuretics, dyslipidemia treated with a statin, and a diagnosis of rheumatic polymyalgia and fibromyalgia. Since 2005, the patient was diagnosed with autoimmune small-fiber neuropathy with negative serology for autoantibodies, but positive skin biopsy, with severe pain poorly responsive to high doses of pregabalin and duloxetine. The patient achieved moderate pain control only during steroid and mycophenolate immunosuppressive treatment.

Until 2004, the patient was treated with MDI according to a basal-bolus regimen, with limited benefit on glycemic control and increased frequency of hypoglycemic events. In 2004, the patient was started on continuous subcutaneous insulin therapy CSII using an external pump with insulin glulisine, but without evident benefits in terms of hyperglycemia control or reduction of the risk of diabetic ketoacidosis (DKA).

For these reasons, in 2007 she underwent a first pancreas transplantation, complicated by thrombosis with organ explant on the seventh day post-surgery. In 2009, a second pancreas transplant was attempted and a combined immunosuppressive therapy with anti-thymocyte globulin (ATG), steroids, mycophenolate mofetil (MMF) and tacrolimus was started. The second transplant was complicated by an intra-abdominal candidiasis treated with fluconazole and hemorrhagic shock due to an injury of the right iliac artery, which underwent ligation and subsequent crossover bypass to treat ischemia of the right lower limb.

After the second transplant, glycemic control initially improved with a reduction in HbA1c from 75 mmol/mol (9.0%) during the 3 months prior to surgery to 61 mmol/mol (7.7%) in the following quarter. No glucometabolic data before and immediately after the first transplant are available, because they predate the minimum backup of our clinical database.

In 2011, the patient received the first intraportal infusion of pancreatic islets, obtaining an improvement in glycemic control with HbA1c decreasing from 87 mmol/mol (10.1%) before surgery to 45 mmol/mol (6.3%) 3 months after, and a reduction of total daily insulin dose (from 25 IU to 13–16 IU). Good glycemic control was maintained until the second pancreatic islets infusion (HbA1c from 58 mmol/mol – 7.4% – to 40 mmol/mol – 5.8% –) in 2012, followed by discontinuation of exogenous insulin. Unfortunately, in 2014 the patient showed a deterioration of glucose control because of loss of pancreatic islet function and resumed exogenous insulin with CSII.

Despite the frequent rotation of the infusion set insertion site, and the avoidance of lipodystrophic areas, and compliance with appropriate catheter replacement times, the patient continued to experience a high glucose averages and a wide glycemic variability, possibly due to erratic SC insulin absorption.

In 2016, the patient underwent several cycles of continuous intravenous insulin infusion due to persistent hyperglycemia and DKA despite CSII treatment (HbA1c 78 mmol/mol - 9.3%). Intravenous (IV) infusion improved glucose profile, with a resolution of ketonemia. A multiple daily insulin regimen was restore (glulisine and degludec). Considering the severe SC insulin resistance and previous therapeutic failures, since 2017, the patient has been re-listed for isolated pancreas transplant.

In order to assess the issue of subcutaneous insulin resistance, several serological tests for autoimmune markers were performed, with negative results, except for a mild and fluctuating positivity for anti-insulin antibodies in a patient not insulin naïve.

Until 2018, the patient monitored her blood glucose levels through capillary testing (SMBG) due to a persistent lack of accuracy in the glucose data from several interstitial sensors tested, both those with enzymatic reaction using glucose oxidase and implantable sensor with fluorescence (Eversense® by Ascensia Diabetes Care).

Considering her brittle diabetes and the severe SC insulin-resistance, CIPII was evaluated as the therapeutic option to improve diabetes control and QoL. In October 2019, after a multidisciplinary consultation, the patient underwent surgery under general anesthesia via laparotomy for the placement of a DiaPort system in the lower left abdominal area. CIPII was started using Insuman Infusat® via an insulin pump (Accu-Chek® by Roche Diabetes Care, Inc.) connected to DiaPort through a catheter. The total basal dose administered was 25–26 IU/day, and the bolus doses were around 20 IU/day (total daily dose of 1,5 IU/kg). In the same time, patient started using a real-time continuous glucose monitoring (rt-CGM) with an external interstitial sensor (Dexcom G6® by Dexcom).

**Figure 1:**
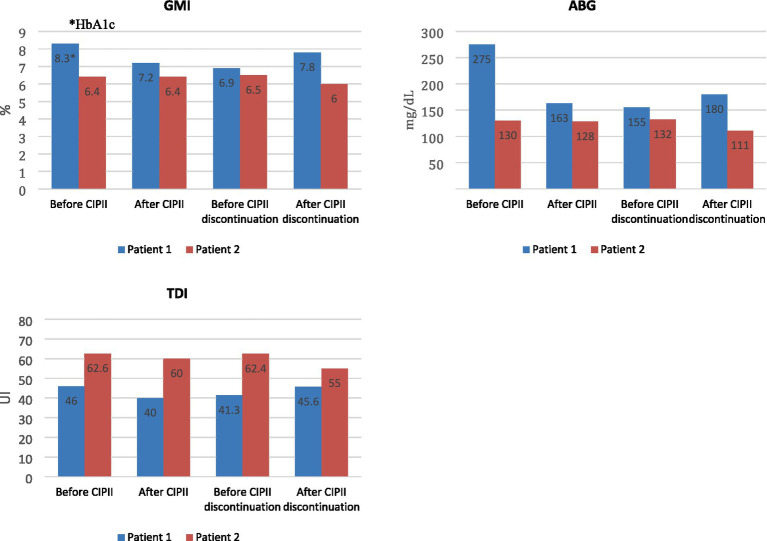
Changes in HbA1c or GMI, ABG and TDI dose in Patient 1 and Patient 2 before and after starting CIPII, and before and after CIPII temporary discontinuation. Prior CIPII implantation, HbA1c value was used as GMI, unavailable, for Patient 1. HbA1c, glycated hemoglobin; ABG: average blood glucose; GMI, glucose management indicator; TDI, total daily insulin dose; CIPII, continuous intraperitoneal insulin infusion.

We report collected data on glycemic control (Figure 1) and rt-CGM metrics 3 months before and after CIPII (Table 1).

**Table 1:** Patient 1 (above) and Patient 2 (below) 3-month glycemic metrics with SMBG/rt-CGM during CSII insulin therapy and 3-month rt-CGM data after starting CIPII, and 3-month glycemic metrics before and after CIPII discontinuation and returning on MDI/CSII.

Treatment: from to	CSII+SMBG July 24, 2019 October 24, 2019	CIPII+CGM November 24, 2019 February 24, 2020	CIPII+CGM February 15, 2023 May 15, 2023	MDI + CGM June 15, 2023 September 15, 2023
TIR 70–180 (%)	14	66	66	36
TAR>180 (%)	-	25	24	50
TAR>250 (%)	-	8	8	13
TBR < 70 (%)	7	1	1	1
TBR < 54 (%)	-	1	1	1
TITR 70–140 (%)	-	39	43	36
TAR>140 (%)	-	60	50	63
CV (%)	46	34	27	39

Treatment: from to	CSII+CGM February 5, 2022 May 5, 2022	CIPII+CGM May 5, 2022 August 5, 2022	CIPII+CGM January 27, 2023 April 27, 2023	CSII+CGM May 27, 2023 August 27, 2023
TIR 70–180 (%)	78	94	90	84
TAR>180 (%)	11	5	8	6
TAR>250 (%)	1	0	0.5	0
TBR < 70 (%)	8	1	1	8
TBR < 54 (%)	2	1	0.5	2
TITR 70–140 (%)	61	71	62	70
TAR>140 (%)	35	28	35	20
CV (%)	33	24	26	34

SMBG, self-monitoring blood glucose; rt-CGM, real-time continuous glucose monitoring; CSII, continuous subcutaneous insulin infusion; CIPII, continuous intraperitoneal insulin infusion; MDI, multiple daily (insulin) injection.

In 2021, due to occlusion of the intraperitoneal catheter, the patient developed DKA requiring initially IV insulin therapy, and then MDI. In the same year, the patient experienced several episodes infections at the site of implant that were treated with oral antibiotic (amoxicillin 1 g *ter in die*), and in March 2022 the DiaPort system was replaced with beneficial effect on glycemic control (HbA1c from 60 mmol/mol – 7.7% – to 50 mmol/mol – 6.7% –).

In May 2023, Insuman Infusat® production was temporarily discontinued, prompting us to switch our patient back from CIPII to SC insulin with multiple daily injections (Apidra® and Tresiba®). We retrospectively analyzed glucose outcomes during this switch in insulin route. At the time of CIPII discontinuation, the patient experienced a period of dissatisfaction with SC insulin therapy and poor compliance with SMBG, which caused extreme glycemic variability and an increase in average blood glucose (ABG) (185 ± 77 mg/dL DS) as showed in Figure 1.

At the beginning of 2024, Insuman Infusat® was available again, insulin therapy was restarted using external insulin pump connected to DiaPort, with an immediate amelioration of glucose control and glucose variability (time in range - TIR 81%; time below range - TBR 1%; time above range - TAR 18%; glucose management indicator - GMI 6.8%; variability coefficient - CV 38%).

In the last year, rt-CGM data confirmed a good glycemic control with TIR 79%, TBR 2%. HbA1c level was stable at 48 mmol/mol (6.6%) and TDI dose was of about 40 IU/day, with no notable changes between SC and IP insulin treatment (Figure 1).

Since then, the patient has continued insulin therapy with the DiaPort System and has maintained good glucose control, with no severe hypoglycemia, a more controlled glucose variability, and a greater satisfaction compared to SC insulin therapy. However, Insuman Infusat® may be discontinued from production in the near future, raising the question of which technology to use to ensure control of glucose variability while mitigating the SC insulin resistance experienced by this patient.

The second patient (Patient 2) is a 58 year-old Caucasian woman, with T1D since age 25, treated using external insulin pump (Accu-Chek®) with aspart insulin since 2009. Her diabetes was complicated by retinopathy. She had a history of hypertension and dyslipidemia treated with statin, migraine with aura treated with topiramate, and hypothyroidism due to chronic thyroiditis. She had also undergone surgical removal of a fibroadenoma from her left breast and a pleomorphic adenoma from her left submandibular gland.

Despite the frequent rotation of infusion set insertion sites, CSII caused severe lipodystrophy in the abdominal and gluteal regions. Due to the subsequent impaired insulin absorption and frequent severe hypoglycemia events, in May 2022 patient was referred to our unit to implant a DiaPort system for CIPII therapy using an external insulin pump (Accu Chek®). She was using an rt-CGM (Dexcom G6®).

As shown in Figure 1, the patient had adequate glucose control (GMI 6.4%) and the ABG (130 mg/dL) already before starting therapy with the DiaPort system. However, she had experienced several severe hypoglycemic events because of hypoglycemia unawareness. The switch to IP insulin delivery not only reduced the frequency of hypoglycemia (<70 mg/dL), but also significantly improved glycemic variability (CV 33% vs. 24%) as reported in Table 1.

In April 2023, because of the temporarily discontinuation of Insuman Infusat® production, the patient returned from CIPII to CSII through the same insulin pump and same rt-CGM. This change in route of insulin delivery increased glycemic variability and the time below range (Table 1).

Currently, the patient maintains adequate glycemic control with a GMI of 6.1% in the last 3-months (TIR 70–180 mg/dL: 89%), a reduced glycemic variability (CV: 30.9%), and no severe hypoglycemia (TBR < 54 mg/dL: 1%).

Since January 2025, the patient has been on the surgical waiting list for the removal of DiaPort system in light of the possible upcoming decommissioning of the device and unpredictable availability of Insuman Infusat®.

## 3 Discussion

**Figure 2:**
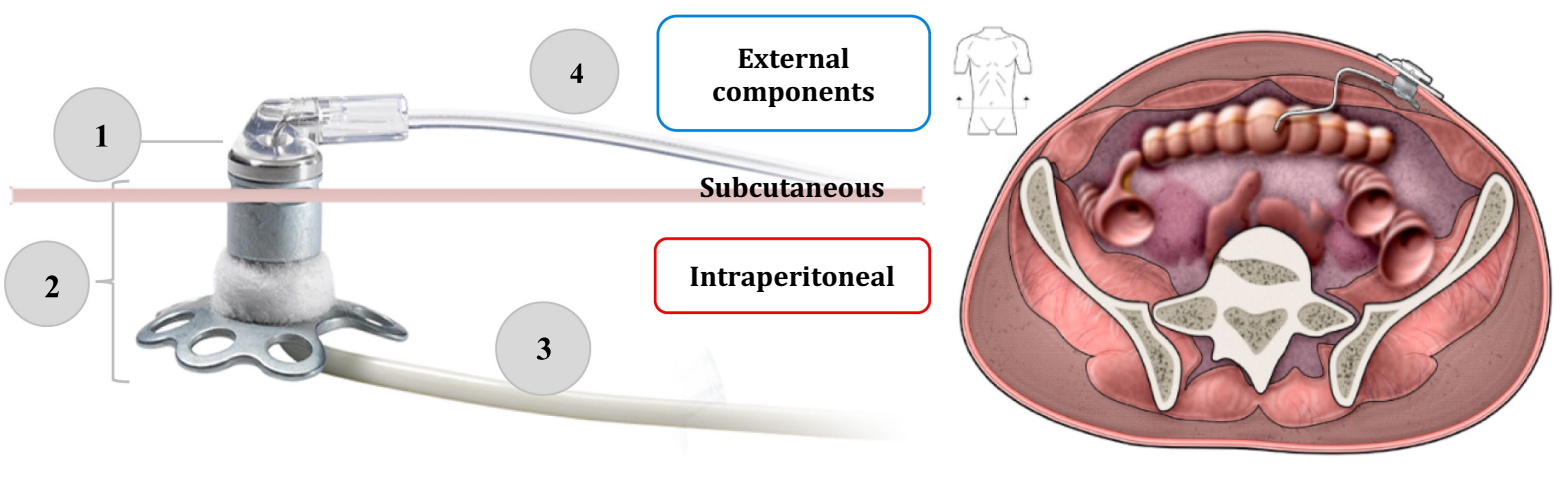
On the left side, the components of the Accu-Chek DiaPort intraperitoneal insulin infusion system: (1) membrane (2) top of the port with flower-shaped plate and a polyester felt band (3) intraperitoneal catheter (4) stainless steel ball cannula infusion set. On the right side, the final implantation site of the device. Adapted from: Accu-Chek® DiaPort system guide use manual (19). Adapted with permission from the Medical Excellence Manager and the Strategic Marketing Manager NPC of Roche Diabetes Care Italy S.p.A. Licensed under Roche Diabetes Care Italy S.p.A. Source: https://www.accu-chek.no/sites/g/files/iut421/f/accu-chek_diaport_handbook_for_port_users_0.pdf.

CIPII has been employed for treatment of diabetes since the 1980s as a preferred option for patients with T1D presenting severe SC insulin resistance (3). CIPII can be implemented either by the implantation of programmable pumps or through a percutaneous port connected to an external insulin pump. Currently, CIPII involves the use of percutaneous access devices through the only system approved in Europe Accu-Chek® DiaPort by Roche Diabetes Care, since 2016. It is a percutaneous titanium port with a catheter placed in the peritoneal cavity, and connected to an insulin pump by a stainless steel ball cannula infusion set as showed in Figure 2. The top of the port protrudes above the surface of the skin about 5 mm, and a flower-shaped plate and a polyester felt band are placed under the skin to stabilize the port (2). The port is implanted during a minor surgery, using general anesthesia. Insuman Infusat® (soluble human insulin by Sanofi Aventis) is the only insulin formulation recommended for use with the Accu-Chek® DiaPort, but, although registered, it is not commercially available in Italy and need to be imported. Fast-acting insulin analogues are not usable in the DiaPort catheter because of their physical instability in this environment, which prompts insulin aggregation in the catheter lumen.

### 3.1 CIPII: a more physiologic insulin delivery route

In cases of abnormal SC insulin absorption, CIPII allows a faster blood glucose normalization after meals with a reproducible and more predictable insulin profiles than SC route. In fact, insulin can be detectable in blood already within a minute after intraperitoneal (IP) administration. Most IP insulin is absorbed via the portal vein, enhancing hepatic uptake (60–80%) and reducing peripheral insulin levels (20–40%) compared to intravenous administration. Lower peripheral insulin levels may also help preserve glucagon response and hepatic glucose production during hypoglycemia or exercise (7). In contrast, SC insulin administration by MDI and CSII therapy may lead to unpredictable fluctuations in blood glucose concentration because of different reasons: the lag time to insulin action after SC injection, the insulin formulations, and the differences between injection sites, and changes to the injection sites. The main differences between SC and IP insulin delivery routes are summarized in Table 2.

**Table 2:** Differences between SC insulin route (through MDI or CSII) and IP insulin infusion.

Route of insulin administration	SC insulin injection (MDI or CSII)	Intraperitoneal insulin infusion (CIPII)
Delivery site	into the SC fat tissue (usually abdomen, thighs, upper arms, gluteus), via insulin pens or external insulin pumps	into the peritoneal (abdominal) cavity, via catheter connected to an external pump (or via an implantable pump)
Insulin absorption and kinetics		
Absorption speed	slower and more variable	faster and more predictable
Portal insulin delivery	mainly systemic absorption; minimal hepatic first-pass	mimics physiological insulin delivery via portal vein
Onset and peak	delayed onset and longer duration	closer to natural insulin action
Glycemic control		
Postprandial control, Hba1c reduction	less effective postprandial control, adequate but may require fine-tuning and close monitoring	improved due to faster absorption, can be more pronounced in some patients
Risk of hypoglycemia	higher risk if not carefully adjusted	lower risk due to better glucose-insulin timing
Clinical use and indications		
Indications	all types of diabetes requiring insulin	unstable T1D not well controlled with SC insulin therapy
Availability	widely available	limited; requires specialized centers
Invasiveness	non-invasive/minimally invasive	invasive, requiring surgical procedure
Cost and manteinance	generally lower cost	high initial and maintenance cost, requiring surgical replacement if necessary
Insurance coverage	commonly covered	limited in many countries
Risks and complications		
Infection risk and device complications	risk of lipohypertrophy and local irritation (MDI), pump site failure and occlusions (CSII)	risk of peritonitis and pump site infections; catheter blockages

SC, subcutaneous; MDI, multiple daily (insulin) injection; CSII, continuous subcutaneous insulin infusion; CIPII, continuous intraperitoneal insulin infusion.

CIPII can be a valid treatment option in T1D patients with the following characteristics:

- Frequent severe hypoglycemia during SC insulin therapy.
- Hypoglycemia unawareness.
- SC insulin resistance.
- Unachieved HbA1c targets on SC insulin therapy (or only at the expense of an increased frequency of hypoglycemic episodes).
- Marked insulin resistance with high insulin requirement.
- Lipoatrophy associated with SC insulin administration.
- Insulin-associated lipohypertrophy not avoided or limited by adequate injection site rotation.
- Skin disorders interfering with SC insulin administration (for example, inflammatory skin disease due to a type 3 immune complex reaction-Arthus reaction-localized).
- Marked fluctuations in glucose levels and insulin requirements with SC insulin therapy.
- Allergies to materials required for SC insulin administration (for example, steel, nickel or adhesive plasters).

CIPII is not indicated in cases of patients with high circulating levels of insulin autoantibodies, poor therapy compliance, evidence of psychiatric conditions, gastrointestinal disorders (e.g., colon diseases, peritoneal adhesions), and unsuitability for external pump use in CSII (1). Some conditions that would require caution in use are also: severe impairment of liver function, impaired immune response or receiving concomitant drug infusion via intraperitoneal (e.g., chemotherapy) or by (continuous ambulatory peritoneal dialysis - CAPD).

The role of circulating anti-insulin antibodies in CIPII therapy is controversial. A marked and sustained elevation of their levels during CIPII has been reported, associated with the formation of immunogenic aggregates with a possible delay in insulin action and hyperglycemia, especially postprandial, or unpredictable hypoglycemia (8). However, other studies found no significant effects on glycemic control from increased anti-insulin antibodies, also considering the clinical benefits of CIPII on glucose variability and diabetes-related complications (9).

Here, we give an overview of the beneficial aspects of CIPII and main evidences supporting this system as a part of treatment portfolio for T1D. Some clinical trials have compared CIPII therapy with SC insulin administration by multiple injections or CSII in term of HbA1c improvement and reduction of hypoglycemic events (5, 10). The main results of these studies are summarized in Table 3.

**Table 3:** Clinical studies comparing CIPII and SC (MDI or CSII) insulin therapy in T1D patients concerning HbA1c, glycemic variability and hypoglycemic events.

Study	Study type	Participants (T1D)	HbA1c	Hypoglycemia	Glycemic variability	TDI dose	QoL / Treatment satisfaction
Haardt et al. (10)	RCT crossover	10	↓HbA1c compared to SC from 8.5 to 7.2%	↓ mild hypoglycemic events (5.7 vs. 10.0/month, *p* = 0.02)	improved with CIPII	not significantly different	↑ treatment satisfaction and QoL
Liebl et al. (4)	RCT crossover (multicenter)	24	↓ HbA1c from 7.4 ± 7.0 (*p* < 0.01)	↓ mild and severe hypoglycemia	not specifically reported	↓ TDD by ~25%	↑ satisfaction with therapy
Logtenberg et al. (5)	RCT crossover (16 months)	24	↓ by ~0.8% (−0.76, 95% CI –1.41 to −0.11)	no significant reduction in mild or severe events (mild: 3.5 vs. 4.0/week, *p* = 0.13; severe: NS)	time in euglycemia ↑ by ~11% compared to SC	not significantly different	not reported in primary paper
Logtenberg et al. (6)	extension on above, treatment satisfaction and HRQOL	24	no major difference, ↓ by ~0.8%	comparable hypoglicemic rates	↓ coefficient of variation by 4.9% (*p* < 0.05)	not reported	↑ treatment satisfaction and HRQoL
Renard et al. (15)	pilot feasibility of AP with IP insulin and sensor in T1D	10	↓ HbA1c in small cohorts	better postprandial control, ↓ hypoglycaemia risk (TIR)	improved time-in-range	not reported	QoL not assessed in pilot
Van Dijk et al. (11)	6-year follow-up of 2014 RCT	20	stable, sustained HbA1c	↓ severe hypoglycaemic episodes	Not specifically reported	maintained reduction	↑ HRQoL, long-term satisfaction
Van Dijk et al. (13)	case–control study (26 weeks)	~176	no major difference	comparable hypoglicemic rates	↓ coefficient of variation by 4.9% (*p* < 0.05)	not reported	not reported
Liebl et al. (2)	prospective, observational	117 (CIPII registry)	↓ HbA1c	↓ hypoglycaemic events	↓ in some patients	↓ vs. baseline	↑ satisfaction
Dassau et al. (16)	pilot clinical study with closed-loop	6 (short-term)	↓ postprandial glucose spikes	↓ hypoglycemia after meals	more physiological insulin profile	not reported	potential for future closed-loop application
Dirnena-Fusini et al. (12)	systematic review and meta-analysis	32 studies included	↓ HbA1c by 0.61% vs. CSII	↓ severe hypo/hyperglycemia	↓ glycemic variability	↓ TDD vs. SC	↑ satisfaction reported

↑, increase; ↓, decrease; HbA1c, glycated hemoglobin; TDI, total daily insulin dose; QoL, quality of life; HRQoL, health-related quality of life; CIPII, continuous intraperitoneal insulin infusion; SC, subcutaneous; CSII, continuous subcutaneous insulin infusion.

CIPII resulted in a significant clinical benefit with a great reduction in HbA1c levels in Patient 1, approximately of −6%, in the 3 months following the placement of the DiaPort. Therefore, the advantage of this insulin delivery route was documented by a TIR improvement from 14 to 66%, and a reduction of over 100 mg/dL in ABG level (as showed in Figure 1). During the temporary return to multiple SC injections, a further worsening of the ABG and GMI as a surrogate of HbA1c, a significant reduction in TIR and in TITR (time in tight range, 70–140 mg/dL), and an increase in TAR, also confirmed the benefit of CIPII on glycemic control in Patient 1 (Table 1).

In the case of Patient 2, glycemic control in terms of HbA1c was already satisfactory prior to the transition to CIPII (HbA1c 6.4% in the quarter before the DiaPort implantation). However, this variable could have been influenced by the presence of a high frequency of hypoglycemic episodes (<70 mg/dL), often unnoticed, related to erratic SC insulin absorption despite an optimal basal doses on CSII. Starting of CIPII, reduced hypoglycemic events (TBR from 10 to 2%) in this patient and significantly limited the need for carbohydrate intake corrections (Table 1). For Patient 2, returning to SC insulin therapy caused a new increase of time spent in hypoglycemia (Table 1) and a decrease in both the ABG and HbA1c levels (as showed in Figure 1).

Many observational studies also found a lower incidence of hypoglycemia (with reduction up to 83%), along with an improvement of HbA1c using CIPII versus SC insulin route (2, 11), as reported in Table 3. Moreover, the incidence of severe hypoglycemia with CSII was more than twice the one on CIPII (4). A more recent systematic review and meta-analysis highlighted that CIPII improved overall glucose control by a significant reduction of HbA1c and fasting insulin levels versus CSII therapy in unstable T1D (12). Among primary outcomes, the frequencies of hypo- and hyperglycemia during CIPII were reduced, despite the TDI dose remained unchanged compared to that during CSII. It is likely because of insulin concentrations peaked faster and returned to baseline levels more quickly after the administration of insulin boluses during CIPII treatment (12).

CIPII is able to improve glucose variability (up to 5%) compared to SC administration, regardless of the type of glucose monitoring used (2). In fact, both studies conducted prior to the availability of rt-CGM and those conducted using rt-CGM have ultimately shown lower glucose variability (CV) with CIPII compared to SC insulin administration (MDI and CSII) (13). Indeed, glycemic variability is not only a predictor of hypoglycemic events and other diabetes-related complications, but it is also a very relevant aspect of glycemic control, and an additional independent risk factor for diabetes-related complications (14).

In our patients, CIPII led to an improvement in the glycemic CV based on metrics derived from both capillary SMBG and interstitial rt-CGM. As expected, the temporary return to standard SC insulin therapy caused a worsening of glycemic variability above the acceptable threshold (≤ 36%).

Beyond glycemic control, CIPII treatment positively affects QoL with a significant improvement of self-reported general QoL on the SF-36 questionnaire and treatment satisfaction in comparison with CSII therapy in several crossover and randomized trials (RCTs) (6), as showed in Table 3. In both the cases we have presented, the use of the DiaPort system improved the QoL and the patients’ attitude toward a more active management of diabetes and insulin therapy. Notably, Patient 1, who had previously not accepted the use of rt-CGM, started using them after the DiaPort placement, leading to further benefits in glycemic control. In fact, for Patient 1, an only slight increase in CV was observed during the period when CIPII was interrupted, and it can be hypothesized that maintaining glycemic monitoring through rt-CGM played an important role.

Therefore, encouraging results have emerged from a non-randomized study on 10 T1D patients sequentially treated with a fully automated closed-loop by SC insulin delivery and by intraperitoneal insulin route (15). Use the IP insulin delivery (DiaPort) plus artificial pancreas (AP) resulted in better glucose control in term of percentage of TITR (70–140 mg/dL), HbA1c and hypoglycemic events (16).

### 3.2 Complications and costs of CIPII

The most reported complications of CIPII were infections at the port site (0.025 events/patient/month), and replacement of several catheters due to occlusions (0.02 events/patient/month) (17). Our clinical experience is in line with the available literature data.

CIPII using implantable programmable pumps was very promising. However, several problems, including reimbursement of pump cost, need to refill the reservoir in an outpatient setting, high development costs, catheter blockage, pump pocket infections, etc., have made this approach increasingly rare. An IP access combined with an external pump allows more flexibility and independence for patients, although issues like catheter blockage and site infections are still unsolved. Therefore, CIPII with external pump enables a shift to closed-loop insulin delivery (15). Unlike current SC-based closed-loop systems, which struggle with postprandial glucose control and delayed insulin action, CIPII offers faster insulin kinetics. This can improve post-meal glucose regulation and reduce hypoglycemia (15). A small study supports its potential, showing near-physiological insulin absorption, quicker glucose normalization, and fewer hypoglycemic events (17).

An important issue of CIPII therapy is its high cost for implantation procedures, substitution of membrane of the port, human insulin importation, with first-year expenses around €31,000 and about €7,500 annually thereafter (about €6,000 more per year than the cost of CSII) (6). Despite the high upfront costs, CIPII may be cost-effective in patients with unstable glucose control by significantly reducing hospital stays and DKA-related admissions (18). The case of Patient 1 documents this benefit, showing a marked drop in hospitalizations and emergency IV insulin treatments after switching to CIPII.

Pancreas or islet cell transplants are, if successful, the only biological treatments currently available to prevent long-term complications in T1D. While potentially effective, their drawbacks such as surgical risks, donor dependency, high rejection rates, and the need for lifelong immunosuppression, limit their appeal. In Patient 1, a pancreas transplant initially improved glycemic control (notable reduction in HbA1c), but failed due to complications. Later islet infusions stabilized glucose levels for several years, even allowing for the temporarily suspension of exogenous insulin, but eventually lost function despite intensive immunosuppression.

## 4 Conclusion

Based on the combined results of improved glycemic control, better QoL and treatment satisfaction, CIPII may be a valid treatment option for T1D patients with inadequate glycemic control or with frequent experience of unexpected hypoglycemic events on standard MDI or CSII therapy. CIPII has clear advantages over SC insulin administration in term of pharmacokinetic and pharmacodynamics properties and has been shown to improve glycemic regulation in T1D patients who failed to reach an adequate glycemic control despite intensive SC insulin therapy.

The cases presented are among the few found in the national clinical landscape and, therefore, represent examples of the applicability of CIPII in selected T1D patients who have a “real” inadequate SC insulin absorption. To this end, it is always useful to exclude in the patient’s clinical history psychological conditions of fragility or impairment that may interfere with the proper management and consistent administration of insulin therapy.

The most significant and encouraging results highlighted by our reports are the improvement in glycemic control based on the reduction of HbA1c, a better glycemic variability, and less frequent hypoglycemic events, including severe ones, not only when starting CIPII, but also after its reintroduction following a temporary suspension. Both the reduction in hospital admissions for DKA and the decrease in severe hypoglycemic events have led to an improvement in the QoL of our patients.

Another possible benefit of IP insulin delivery could be a potential improvement of the performance of AP with the use of a fully automated CL system. At present, only hybrid CL with SC insulin administration are available. In these systems, patients have to inform the system of the amount of carbohydrates ingested, from which the system calculates the bolus of insulin to be administered. The development of a fully closed-loop AP requiring no regular daily intervention by the patient and at the same time maintaining glucose levels in the normal or close-to-normal range remains distant. For this reason, a potential switch from SC to CIPII insulin delivery could help to make this a feasible and clinically more valid therapeutic option in selected patients.

Our aim was not only to describe our clinical experience with CIPII, but also to highlight the need for this insulin delivery system to remain available for centers of excellence, combined with stable availability of compatible human insulin, and to establish a path for accessing this technology even for more peripheral diabetes centers.

The potential discontinuation of DiaPort, and/or the limited availability of compatible insulins, would make the management of diabetes and the control of its acute complications more challenging for T1D patients with very unstable glucose control. The available alternatives, such as transplants and hybrid insulin pump systems with automatic working algorithms, have only a limited potential for these patients, considering that intensive pharmacological therapies in the first case and SC insulin administration in the second one remain important limiting factors.

## Data Availability

The raw data supporting the conclusions of this article will be made available by the authors, without undue reservation.

## Glossary

CIPII: continuous intraperitoneal insulin infusion
T1D: type 1 diabetes
MDI: multiple daily (insulin) injection
CSII: continuous subcutaneous insulin infusion
SC: subcutaneous;
QoL: quality of life
ATG: anti-thymocyte globulin
MMF: mycophenolate mofetil
DKA: diabetic ketoacidosis
SMBG: self-monitoring blood glucose
rt-CGM: real-time continuous glucose monitoring
HbA1c: glycated hemoglobin
ABG: average blood glucose
CAPD: continuous ambulatory peritoneal dialysis
TIR: time in range
TBR: time below range
TAR: time above range
GMI: glucose management indicator
CV: variability coefficient
TITR: time in tight range
IP: intraperitoneal
AP: artificial pancreas
CL: closed-loop
TDI: total daily insulin dose
IU: international unit
IV: intravenous
RCT: randomized controlled trial
